# Give the Babies Water

**Published:** 1887-08

**Authors:** 


### Give the Babies Water.

Dr. Touissaint, in an article in the *Union Medicale de Canada*, calls attention to the fact that milk does not satisfy the thirst of babies. It appeases hunger, but it frequently intensifies thirst, and the author maintains that it is this very thirst that causes healthy children, raised altogether at the breast, to cry so frequently and so violently. We have seen peevish, fretful infants, upon whom all the arts of the nurse and mother had in vain been tried without eliciting a smile, suddenly brighten up at the sight of water, reach eagerly for it, and on obtaining a drink, go off to sleep calmly and contentedly. We quite agree with Dr. Touissaint when he declares that many cases of infantile indigestion would be benefited or cured by giving the little patients a regular supply of water.—*St. Louis Med. and Surg. Journal.*

